# Stable, Ductile and Strong Ultrafine HT-9 Steels via Large Strain Machining

**DOI:** 10.3390/nano11102538

**Published:** 2021-09-28

**Authors:** Osman El-Atwani, Hyosim Kim, Jonathan G. Gigax, Cayla Harvey, Berk Aytuna, Mert Efe, Stuart A. Maloy

**Affiliations:** 1 Materials Science and Technology, Los Alamos National Lab, Los Alamos, NM 87545, USA; hkim@lanl.gov (H.K.); cayla@lanl.gov (C.H.); maloy@lanl.gov (S.A.M.); 2 Center for Integrated Nanotechnologies, Los Alamos, NM 87545, USA; jgigax@lanl.gov; 3 Chemical and Materials Engineering, University of Nevada, Reno, NV 89557, USA; 4 Department of Metallurgical and Materials Engineering, Middle East Technical University, Ankara 06800, Turkey; aytuna.berk@metu.edu.tr (B.A.); mert.efe@pnnl.gov (M.E.); 5 Energy and Environment Directorate, Pacific Northwest National Laboratory, Richland, WA 99354, USA

**Keywords:** nanocrystalline, large strain machining, microtensile, nanoindentation, HT-9 steel

## Abstract

Beyond the current commercial materials, refining the grain size is among the proposed strategies to manufacture resilient materials for industrial applications demanding high resistance to severe environments. Here, large strain machining (LSM) was used to manufacture nanostructured HT-9 steel with enhanced thermal stability, mechanical properties, and ductility. Nanocrystalline HT-9 steels with different aspect rations are achieved. In-situ transmission electron microscopy annealing experiments demonstrated that the nanocrystalline grains have excellent thermal stability up to 700 °C with no additional elemental segregation on the grain boundaries other than the initial carbides, attributing the thermal stability of the LSM materials to the low dislocation densities and strains in the final microstructure. Nano-indentation and micro-tensile testing performed on the LSM material pre- and post-annealing demonstrated the possibility of tuning the material’s strength and ductility. The results expound on the possibility of manufacturing controlled nanocrystalline materials via a scalable and cost-effective method, albeit with additional fundamental understanding of the resultant morphology dependence on the LSM conditions.

## 1. Introduction

Fourth generation nuclear (Gen IV) fission reactor designs are currently explored and developed for attaining ultimate goals of sustainability, efficiency, and safety. These new designs require novel structural materials that can withstand higher radiation doses, temperatures, and mechanical stresses when compared to current light water reactors [1,2]. Therefore, the search for advanced nuclear materials is paramount and a priority to achieve success in Gen IV reactors. Ferritic/Martensitic (F/M) steels are known to be primary candidates as structural and cladding materials for Gen IV reactors given their documentation over a long period of time in research [3]. Some of these steels are the first generation F/M steels (HT-9 with 12% Cr, 1% MoVW) and the second generation modified steels (e.g., Grade 91 with 9% Cr and 1% Mo) [4]. These steels exhibit advantages over austenitic steels in terms of void swelling and physical properties (reduced thermal expansion coefficient and improved thermal conductivity) [4,5,6,7]. While these steels provide excellent resistance to atmospheric corrosion and many organic media, their utilization is however limited to around ~560 °C due to thermal creep and associated loss of strength at higher temperatures [1]. This is a key concern, given that the use temperature of fuel cladding materials in the future fleet of reactors is expected to approach 650–700 °C [8]. Another challenge, for example with HT9, is embrittlement (loss of fracture toughness) that occurs due to defect cluster hardening after low doses of irradiation at low temperatures (below 400 °C) [9]. Therefore, there is still a need for an advanced generation of steels for Gen IV reactors [1].

Fundamental experimental and theoretical studies on materials have demonstrated higher radiation resistance of nanostructured materials (materials with grain sizes in the nanoscale regime) [10,11,12,13]. These materials have a high density of grain boundaries that can act as defect sinks and possibly minimize degradation in the mechanical properties of irradiated nuclear materials [14,15,16,17,18,19,20]. Along the same line of research, recently developed nanostructured ferritic alloys [21,22,23], developed from oxide dispersion steels strengthened (ODS) steels with ultrafine grains and 2–5 nanometer oxide particles (e.g., 14YWT with composition of Fe-14Cr-3W-0.4Ti-0.3Y_2_O_3_), represent a new generation of advanced structural nuclear materials. These steels have high recrystallization temperatures [24], high temperature strength, high creep strength, low ductile to brittle transition temperature, and excellent radiation resistance [25].

However, these steels have several drawbacks to overcome. ODS steels are processed via powder metallurgy method with mechanical alloying for an extended time (40 h for attritor milling) [25]. The scalability of these steels and the cost of scale up production has been an issue. Furthermore, the composition of powders, processing methods, and fabrication parameters including mechanical alloying via ball milling and possible contamination affect the microstructure and, thus, influence the mechanical properties and radiation resistance [26]. The usual bimodal distribution of grains can also create anisotropy in the mechanical properties [27]. A recently developed nanostructured ODS steel, OFRAC [28], was manufactured as a cladding material with improved creep resistance to other steels (e.g., commercial HT-9), but studies are still ongoing regarding their radiation resistance and drawbacks from mechanical alloying could still be an issue. Other steels, manufactured in nanocrystalline form via mechanical alloying and powder metallurgy have shown to possess ultra-strength, thermal stability, and radiation tolerance [20].

**Figure 1:**
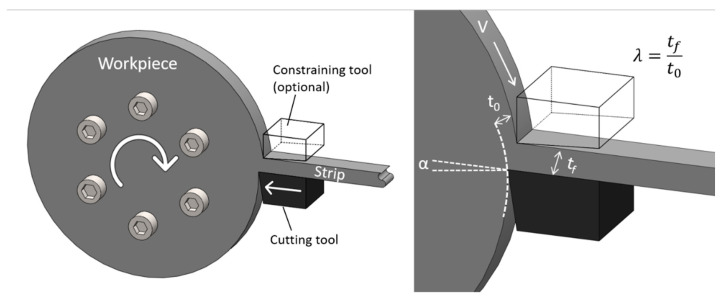
Schematic of the LSM process used to manufacture samples A&B.

The question that arises is whether nanostructured steels can be manufactured using other methods, with enough thermal stability to survive harsh nuclear reactor environments. Severe plastic deformation methods were shown to successfully produce nanostructured metals [29]. The Large Strain Extrusion Machining (LSEM) process [30,31], is a cost-effective method that can produce large thin metal sheet forms directly from a coarse-grained feedstock without the need for elevated temperature processing. Figure 1 shows one configuration of LSEM, where a thin continuous strip is directly “peeled” away from the surface of a bulk metal feedstock by simultaneous cutting and extrusion. This concept, producing thin cross-section metal products via controlled material removal is fundamentally different to conventional processes such as rolling, in that the shape change is accomplished in just a single stage of deformation. It is also important to note that, unlike conventional machining, the thickness of the strip (chip) exiting the cutting tool can be controlled using an additional constraining edge that is placed directly across the cutting edge (Figure 1). Some unique features in LSEM when compared to most other deformation processes include: (1) ability to impose extreme plastic strains (up to five) in a single deformation step; (2) high rate deformation which enables large adiabatic heating in the deformation zone; and (3) large hydrostatic pressures in the range of 2–4 k (with k being the shear yield stress of the material) [32]. These characteristics are especially beneficial for processing metals that are prone to processability or material failure issues, without extensive need for multiple heat treatments and numerous deformation passes. LSEM was successfully used to manufacture nanocrystalline materials from Al, Mg, W, Ni, Fe-Si, steels, and alloys [30,31,33].

Here, we demonstrate the use of Large Strain Machining (LSM), LSEM without a constraining edge, to manufacture nanostructured HT-9 steel (processed from ultrafine HT-9) with enhanced thermal stability, mechanical properties (strength comparable to tungsten) and decent ductility despite the grain size being the in nanocrystalline and ultrafine regime. It is also shown that post-LSM annealing can result in recrystallization but a formation of stable ultrafine grains. This material is considered to be a new generation of nanostructured steels capable of withstanding severe nuclear reactor environments.

## 2. Materials and Methods

HT-9 discs were purchased from American Elements^®^ (Los Angelos, CA, USA). The elemental compositions of the discs are in Table 1.

One disc was heated to 1040 °C with a slow ramp rate and then slowly cooled, and one disc followed the same treatment but then was tempered at 760 °C for ~3 h. LSM (schematic shown in Figure 1) was then performed on the discs with no external heating and one sample was generated from every disc (sample A is made from the annealed and slow cooled disc and sample B is made from the tempered disc, see Table 2). The conditions of LSM and the output parameters are presented in Table 2.

The effective strain imposed on the strips can be determined by idealizing the deformation zone as a single shear plane [34]. The effective strain can then by calculated by:

(1) $$\gamma =\sqrt{3}\bar{\varepsilon }$$

where γ is the shear strain. γ can be found by:

(2) $$\gamma =\frac{\lambda }{cos\alpha }+\frac{1}{\lambda cos\alpha }-2tan\alpha$$

where α is the tool rake angle and λ is the chip or strip thickness ratio or t_c_/t_0_ where t_c_ and t_0_ are the final and the uniformed chip thicknesses, respectively.

The relation of the strain rate to the deformation speed is given by: $\dot{\bar{\varepsilon }}$ ~ $\bar{\varepsilon }$V/Δ where V and Δ are the deformation speed and the thickness of the deformation zone, respectively [31].

The deformation zone temperature (ΔT) can also be estimated using the shear plane model which uses specific shear energies and velocities as inputs [35]. The input parameters for the ΔT calculations were based on 420 stainless steel properties [36].

Small 3 mm discs were then punched out from the samples, mechanically polished and then electropolished for electron backscattering diffraction (EBSD) characterization. Transmission Electron Microscopy (TEM) (samples were also prepared via electropolishing. The electropolishing solution was 5% perchloric acid in methanol and the electropolishing was performed at −30 °C. The morphology of the samples was characterized with EBSD (electron energy of 20 keV and step size of 25 nm) and 300 keV field emission TEM. In-situ TEM/annealing was performed within the TEM with a ramp rate of ~25 degrees/min.

Nanoindentation using a Keysight G200 nanoindenter, in the center of integrated technologies (CINT), equipped with a Berkovich tip was performed on polished specimens to a depth of 1000 nm at a strain rate of 0.05 s^−1^. Hardness and modulus measurements were computed using the Oliver–Pharr method, assuming a Poisson ratio of 0.3 [37]. For microtensile testing, small scale tensile specimens were fabricated using a femtosecond laser cutting system and procedure described in previous studies [38,39,40]. Tensile specimens were cut using an output pulse width of 350 fs, wavelength of 1053 nm, repetition rate of 20 kHz, and energy of 10 μJ focused through a 5x objective. The final tensile specimen gauge dimensions were 0.045 mm × 0.07 mm. LSM HT9 samples were fabricated with a gauge length of 0.3 mm. Due to the larger fracture strain of the tempered HT9 sample, the gauge length for this specimen was fixed at 0.15 mm. Two or three tensile specimens were tested for each condition using an in-house designed and fabricated tensile testing system and pulled at an initial strain rate of 2.5 × 10^−3^ 1/s with a constant displacement rate through the test. The average result of the test is then calculated.

## 3. Results

**Figure 2:**
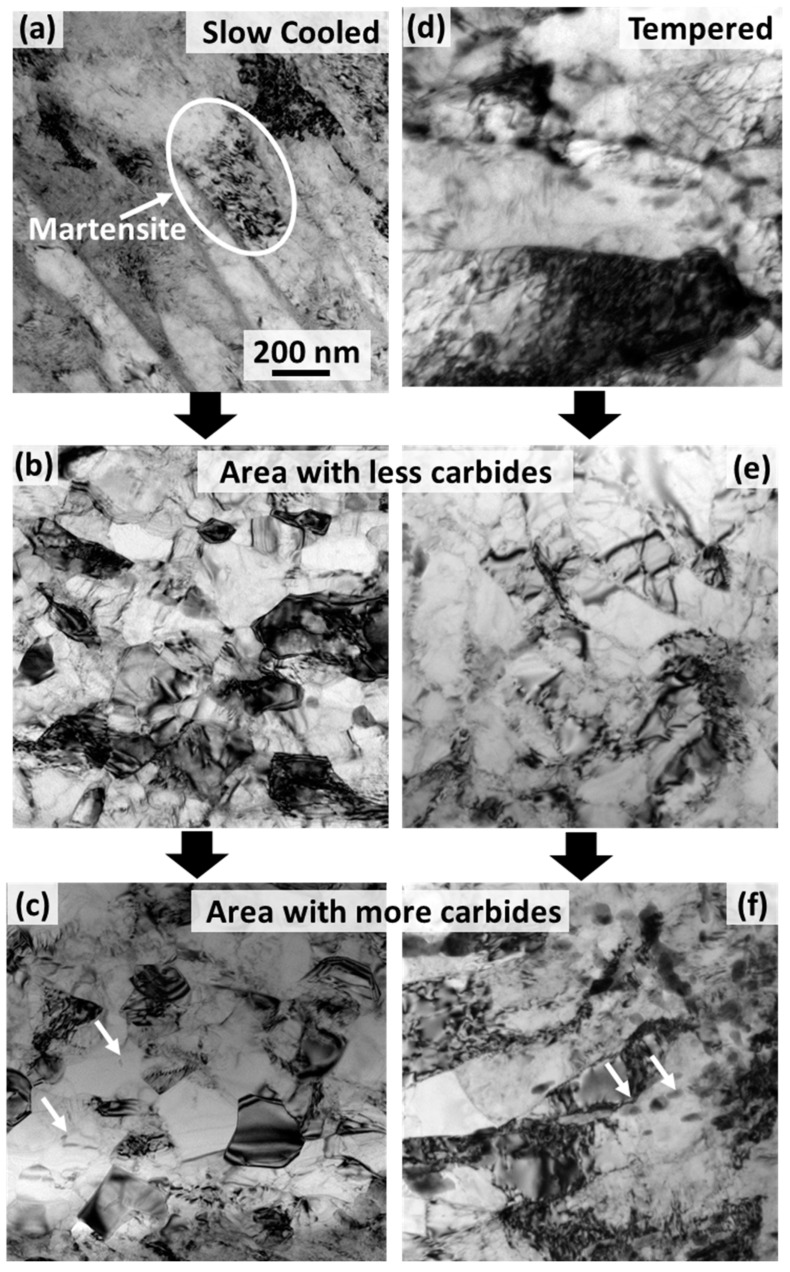
TEM micrographs before LSM (**a**,**d**) and after LSM (**b**–**f**): sample A showing areas without a high density of carbides (**b**) and with high density of carbides (**c**) and sample B without carbides (**e**) and with carbides (**f**). The circle in (**a**) represents a sample of martensitic microstructure in the slow cooled disc before LSM.

**Figure 3:**
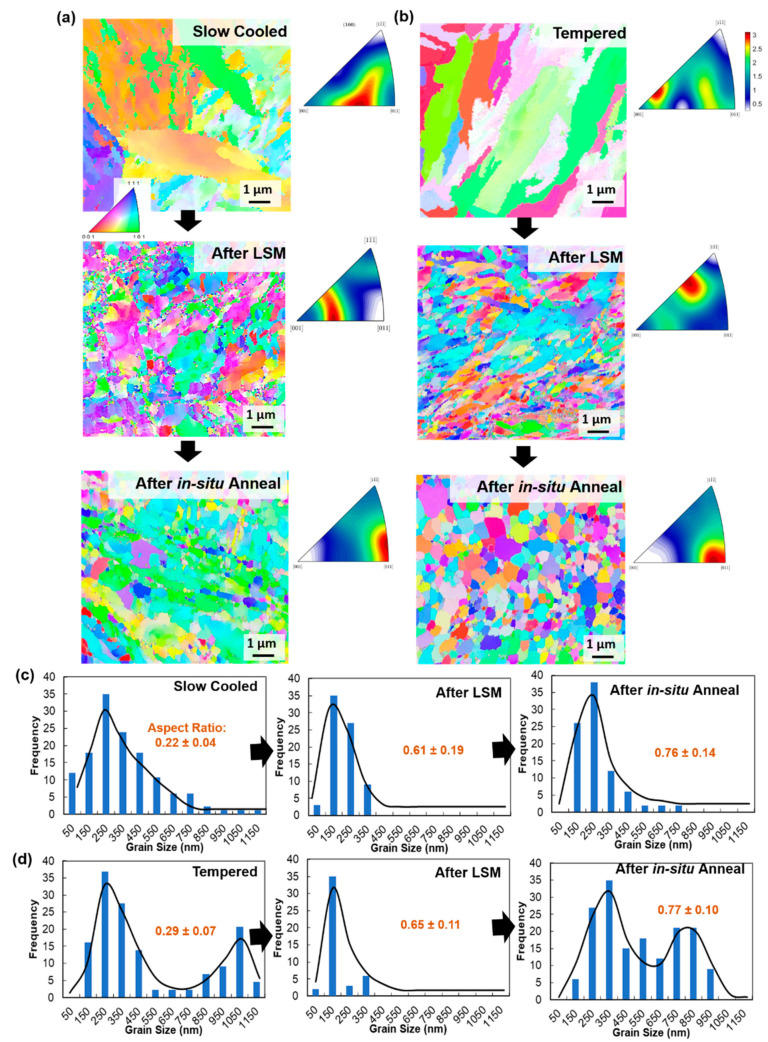
Inverse Pole Figure maps with the corresponding Inverse Pole Figure below, for as is, before and after in-situ annealing of the processed material for (**a**) annealed and slow cooled HT9 (Sample A), and (**b**) tempered HT9 (Sample B). (**c**,**d**) Grain diameter along the larger axes for the samples in (**a**,**b**), respectively, determined from bright-field TEM micrographs. Aspect ratio = diameter minimum/diameter maximum.

**Figure 4:**
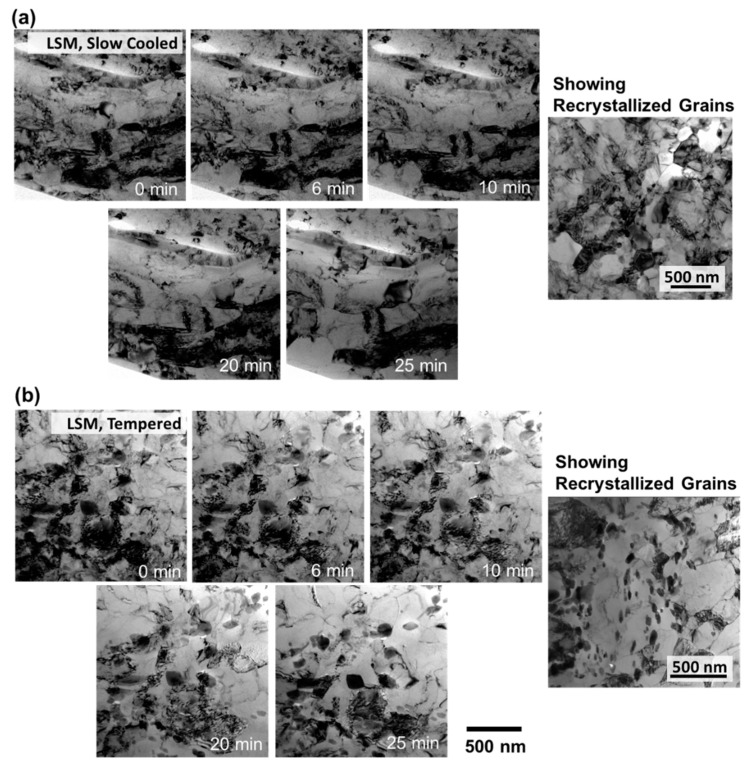
TEM micrographs of in-situ annealing from 25 to 700 °C over 25 min for (**a**) LSM HT9, sample A and (**b**) LSM HT9 annealed, sample B.

The microstructure within the HT-9 disks (as is and annealed) prior to LSEM is shown in Figure 2, Figure 3 and Figure 4. The samples show fine grains with grain boundary carbides in some regions. These carbides were shown previously to be Cr-rich M_23_C_6_ carbides [41]. These carbides were also shown to occur in tempered steels [42]. After LSM, the microstructure within the samples consisted of elongated or equiaxed grains with carbides still located in some regions (Figure 2 and Figure 3). Sample B (LSM sample from the tempered HT-9) showed larger carbides (Figure 2f), which is expected since it came from the tempered HT-9 disc. The EBSD (Figure 3a) shows poorly indexed points from sample A, however, due to high grain boundary strains after deformation. Texture and grain size aspect ratio changes (calculated via averaging several TEM figures), before and after LSM are also evident in Figure 3 for both samples due to the deformation process and the formation of new grains during the LSM process.

To demonstrate the thermal stability of these samples, regions with low carbide densities were selected and the samples were heated to 700 °C. Figure 4 shows the LSM samples A and B heated to 700 °C. Up to 650 °C, no grain growth occurred. Around 700 °C, some regions started to recrystallize. The figures also show recrystallized grains of the samples at 700 °C after 20 min of annealing. These grains were stable, with no growth after about 20 min of annealing at this temperature. Although a growth can further occur at much longer time scale, the microstructure stability for 20 min at 700 °C suggests that any further growth can be slow, and that much longer time scales are needed for grain growth at lower temperatures. Comparison with bulk heating (results not shown here) demonstrated similar results. The EBSD of these specimens are shown in Figure 3.

**Figure 5:**
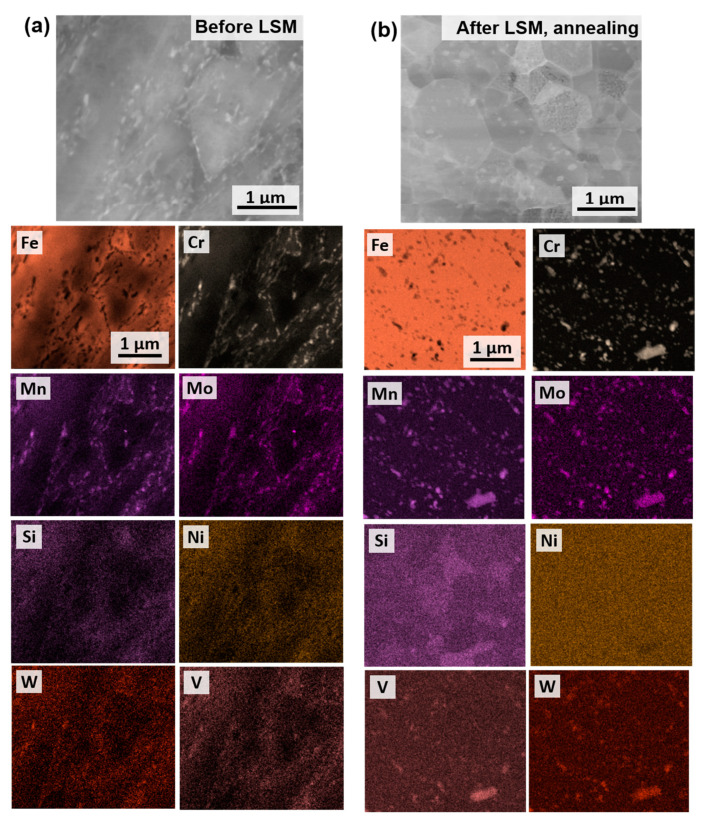
EDX for sample B (**a**) before LSM and (**b**) after LSM and annealing.

Grain size measurement was performed on the samples after LSM and post-annealing. The grain size is determined from bright-field TEM micrographs. Both samples showed a decrease in grain size when compared to the discs before LSM (Figure 3c,d). After annealing to 700 °C, the samples showed grain size increases due to recrystallization, with an overall larger grain size for the sample B compared to the sample A. It is evident that mainly in the recrystallized regions of the samples after annealing, the strain is released (grain boundaries are better resolved in the EBSD), as shown in Figure 3. Elemental analysis was then performed using EDX mapping in the TEM. The EDX was performed on the tempered disc (sample B) before LSM and after LSM and annealing to 700 °C, as shown in Figure 5. The elemental mapping was performed to investigate changes to the carbide composition or possible segregation of elements to the grain boundaries as a reason for grain size stability in the non-recrystallized grains and the thermal stability of the recrystallized grains in the LSM samples. The carbides were shown prior to LSM and post LSM and annealing to have the same elements (Cr, Mo, Mn, V and W) and compositions, and no further segregation of other elements on the grain boundaries occurred. It is also clear that no significant changes occurred regarding the density or size of the carbides during annealing.

**Figure 6:**
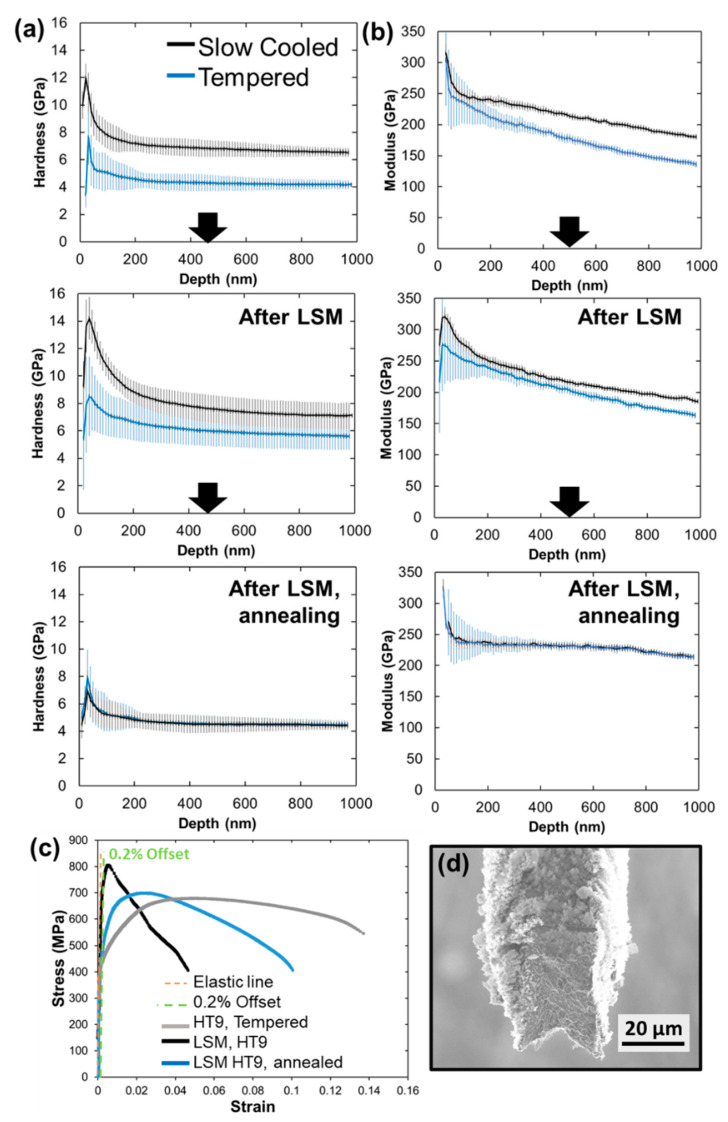
Nanoindentation results showing (**a**) hardness versus displacement and (**b**) modulus versus displacement. Representative plots (taken from one test) of the micro-tensile results (**c**) for the LSM HT9 (sample B) before and after annealing compared to as received HT9. (**d**) Fracture surface for LSM HT9 (sample B) after annealing.

The mechanical properties of the samples were investigated using nanoindentation and microtensile tests. Both were performed at RT. Nanoindentation was performed on both samples A and B. Figure 6 shows displacement vs. hardness and modulus data for samples A and B post LSM and post LSM and annealing. Prior to LSM, the slow disc had higher hardness (~7.5 GPa) compared to the annealed and tempered disk (~4 GPa). After LSM and prior to annealing, sample A showed higher hardness of ~8 GPa while sample B hardness was ~6.25 GPa. After annealing, the values on both samples dropped to ~4.5 GPa which is very similar to the disks’ values before LSM. However, annealing lead to residual stress and martensitic microstructure (e.g., Figure 2a) minimization, and grain boundary recovery. The microstructure, however, remained equiaxed with grains in the ultrafine regime.

To examine the ductility and possible ductility recovery, the microtensile data for only one sample (sample B before and after annealing) is plotted in Figure 6c and is compared to the tempered HT-9 disc before LSM. The LSM sample has nearly 1.75 times the yield strength of the HT-9 disc but with much less elongation. The LSM sample after annealing showed recovery of the elongation and strain hardening, but the yield strength dropped to the value of the tempered HT-9 disc which is consistent with the hardness results from the nanoindentation.

## 4. Discussion

The results in this work demonstrate the possibility of obtaining nanocrystalline grains (elongated vs. equiaxed) from commercial HT-9 discs. The discs were used at different conditions (normalized and slow cooled vs. normalized and tempered) and LSM was performed on the discs at different conditions. The work here demonstrates two conditions (one for each disc) where LSM produced continuous strips. Although different parameters were calculated from the two conditions, the effective strain (shown in Table 1) was nearly the same for both. The temperature rises, due to adiabatic heat generation during deformation were not very different for both discs. However, the grain size, hardness and texture were not the same. This is attributed to differences in discs used for LSM rather than the LSM condition differences. Sample A was taken from a normalized and slow cooled disc of high hardness and martensitic regions (Figure 2a). The increase in hardness for the sample after LSM is ~0.5 GPa, which indicates a small decrease in the grain size. For sample B, the original grain size for the disc prior to LSEM was larger due to tempering at 760 °C. The grain size decrease after LSM (Figure 3) is more noticeable in this sample. Therefore, the increase in hardness after LSM is more noticeable (~2 GPa increase). Since the effective strain, and thus, the material flow is very close for samples A and B, but sample B is formed from a tempered and low hardness disc (compared to the normalized and slow cooled disc used to generate sample A), the higher grain refinement in Sample B is attributed to the initial microstructure. The resultant hardness values were the same for sample A and B post-annealing and were also the same value as the tempered disc before LSM but with less residual strains, which indicates that the hardness of the LSM samples before and after annealing follows the Hall–Petch effect [43] and is grain size dependent. The microstructures, however, are different from the original discs as the grains are equiaxed for sample B or mixed, and equiaxed and elongated (but of much less elongation), for sample A compared to the original discs (Figure 3). The aspect ratio for elongated grains can affect the mechanical properties [44,45] and therefore, direct comparison with the discs prior to LSM is not possible based on grain size only.

Recrystallization for some regions in the LSM was observed in regions with carbides and with less carbides, which indicates that the recrystallization was mainly due to high strains in some regions of the samples. Other regions (Figure 4) did not recrystallize and showed no grain growth. Measurements of local strains in the samples (future work) and correlation with LSM parameters and process can lead to optimization of the parameters of LSM to provide more stable grains. After recrystallization, the grains were stable and the samples showed no further segregation of elements to the grain boundaries other than carbides which are possibly due to re-precipitation after recrystallization or grain boundaries intercepting matrix carbides during growth. The recrystallization leads to strain minimization and the release of grain boundary energy and this provided stability to the grains after further annealing and further support of the possibility of having more stable grains from LSM if strain was to be minimized or distributed, which is possible with performing LSM at high heats.

To demonstrate the ductility and ductility recovery in LSM samples, sample B was chosen since it is formed with equiaxed grains and of bimodal distribution. The microtensile experiment is less affected by grain texture, aspect ratio, and possible non-uniform morphology in the LSM samples compared to nanoindenation. Table 3 provides a summary of the average tensile properties of all samples tested in this study. The decrease in ductility for the LSM samples is accompanied with a high increase in yield strength, which is expected after the grain size refinement and the increase in strain in the LSM sample. These materials have a grain size in the 100–1000 nm regime where the deformation mechanism was previously suggested to be governed by grain boundary shearing promoted by the pile-up of dislocations [46]. Deformation in this regime is well described through the core-and-mantle model [47]. In the model, the grain boundaries are believed to be formed of ledges which can start dislocation formation in the “mantle” layer near the grain boundaries. Dislocations in the “mantle” forms a hardened layer near the boundaries and dominate the plastic flow process, unlike the “core” which undergoes a low work hardening rate. As the grain size decreases in this regime (gets closer to few hundreds of nms, as in the case of the LSM sample), the “mantle” to the “core” fraction increases, causing high yield stress. Although the sample showed some elongation during deformation (Figure 6, Table 3), no strain hardening is observed. Materials subject to severe plastic deformation (SPD) are known to undergo dynamic recovery due to the rise of local temperature [48,49]. The recovery leads to saturation and/or annihilation of dislocations at the grain boundaries, thus leading to a decrease in the work hardening and a necking at the yield stress. Several ultrafine materials formed via SPD have demonstrated this behavior [46,50]. After annealing, however, ductility is mostly recovered, and the yield strength decreased when compared with the LSM sample (prior to annealing) but was similar to the HT-9 disc. An increase in work hardening rate was also shown to occur and the fracture surface (Figure 6d) demonstrated a ductile fracture of 45 degrees to the loading axis. The increase in the hardening rate after annealing is associated with partial recovery of grain boundaries (Figure 2 and Figure 3), which was previously described to occur during low to moderate temperature annealing of SPD materials due to minimization of strain localization [46]. The grain size, after annealing, increased which can describe the drop in the yield strength since materials in this grain size regime still follow the Hall–Petch relationship [43,46]. After the annealing, the sample is also composed of equiaxed grains and of bimodal grain size distribution (Figure 3). A bimodal distribution leads to enhanced strength and ductility where small grains increase the hardness and large grains ensure elongation during deformation and thus, ductility [51,52]. The LSM samples (before annealing) have high strength compared to commercial HT-9 (prior to LSM) and material elongation occurred during deformation. After annealing, larger elongation occurred but hardness decreased. Although the hardness of the discs prior to LSM is similar, the martensitic structure (Figure 2a) of the morphology of the discs prior to LSM would have a large contribution to the hardness. Even the tempered disc is still expected to have martensitic microstructure. Such a microstructure is further minimized in the LSM samples after annealing and the high density of the grain boundaries would have a significant contribution to the hardness of the LSM annealed samples.

Moreover, the grain shape was equiaxed and with much less strain. The equiaxed shape of the grains can lead to enhanced radiation resistance as demonstrated in a previous work on another BCC material [53].

In this work, LSM was used to manufacture nanocrystalline and ultrafine forms of two different HT-9 microstructures. The effective strain was the same in both cases. The results (morphology before and after annealing and hardness before annealing) demonstrated how the initial microstructure can affect the deformation process. Several parameters, however, can be changed during the LSM process and altering the microstructure can be achieved through optimization of the LSM parameters and the initial microstructure. The LSM process should then offer an easy pathway to create controlled and stable nanocrystalline forms of real reactor materials, with very low martensitic fractions, to be investigated at temperature relevant to some Gen IV fission reactors. Nanocrystalline forms of model materials, such as Fe, were previously studied [16]. However, the thermal stability in model materials limited the temperature at which the performance of these nanocrystalline forms can be investigated. The nanocrystalline forms of real reactor materials not only alloy higher temperature studies, but also offer the advantage of understanding the role of the other microstructural elements on several vital factors affecting the overall performance under severe environments such as the matrix and grain boundary sink efficiencies, segregation, thermal stability, and defects behavior.

Possessing outstanding thermal stability, defect sink density and mechanical properties, the performance of these materials under reactor relevant conditions is critical to climb the technology readiness level (TRL). Investigating the irradiation tolerance of these materials at low temperatures where commercial HT-9 suffers from embrittlement, and elucidating how grain boundaries accompanied with alloying elements affect their radiation tolerance should be a next step to qualify these materials for the use in nuclear industry. Since Gen IV reactors are to be operated at temperatures in the range of 350–700 °C, the thermal stability of these alloys demonstrated in this work makes them promising candidates for the nuclear industry. Du et al. [20] has shown outstanding irradiation resistance to grain growth and void swelling in nanostructured austenitic steels at relevant temperatures to nuclear applications. Future works on nanostructured HT-9 can possibly demonstrate similar outstanding irradiation resistance.

Climbing the TRL further depends on whether the process of making these materials is or is not scalable. Bulk nanostructured materials in the form of foils, plates and bars with controlled dimensions were produced via LSEM and thicknesses of up to 2.7 mm (cladding materials are of ~0.6 mm thickness) have been achieved [33]. Moreover, morphology produced LSEM principles can be used as benchmarks for other techniques and can be produced by other materials processing techniques such as friction stir processing [54,55]. The results in this work indicate that further parameter optimization during machining, understanding the deformation process during the low cost, efficient and high throughput LSM process, and the correlation with disc materials used for LSM can lead to nanostructured and thermally stable steels where strength and ductility can be optimized.

## 5. Conclusions

We have performed LSM with two different sets of conditions but with similar effective strain on commercial HT-9 material and achieved nanocrystalline and ultrafine grain sizes with different aspect ratios which depended on the initial state of the HT-9 disc. The nanostructured grains had excellent thermal stability up to 700 °C, at the point where some regions of high strains recrystallized to form grains in the ultrafine regions that were stable during further annealing. EDX mapping performed on one sample (before LSM and after LSM and annealing) demonstrated no additional elemental segregation on the grain boundaries other than the initial carbides (which were available in the HT-9 discs prior to LSM) or the re-precipitated carbides (during LSM) indicating that the high thermal stability of the LSM samples is due to the evident low dislocation densities and strains in the post LSM materials. The hardness of the LSM samples were higher than the initial discs but with lower ductility. After annealing, the hardness decreased but the ductility and the strain hardening recovered. The stable nanocrystalline (prior to annealing) and ultrafine, elongated or equiaxed (post-annealing) microstructure will permit high temperature irradiation resistance investigation of real reactor nanocrystalline materials at prototypic nuclear application (e.g., fission) conditions. The results demonstrate the ability to control the nanocrystalline microstructure with additional fundamental understanding of the resultant morphology dependence on the LSM conditions.

## Figures and Tables

**Table 1:** Elemental compositions of the disc where LSM is performed.

Element	C	Mn	Si	Ni	Cr	Mo	Nb	W	Al	V	N
Composition Range (wt%)	0.17–0.23	0.40–0.70	0.20–0.30	0.30–0.80	11.0–12.5	0.80–1.20	≤0.05	0.40–0.60	≤0.05	0.25–0.35	≤0.001

**Table 2:** LSM input and output parameters for samples A&B.

Samples	t_0_ (mm)	t_f_ (mm)	*λ*	α	*γ*	$\bar{\varepsilon }$	Δ (µm)	V (m/s)	$\dot{\bar{\varepsilon }}$ (1/s)	ΔT (K)
Sample A	0.1	0.155	1.6	−10°	2.6	1.5	100	0.7	1.1 × 10^4^	679
Sample B	0.1	0.265	2.7	0°	3.0	1.7	100	0.7	1.2 × 10^4^	630

**Table 3:** Summary of the average tensile properties of the different specimens tested in the present study.

Specimen	Yield Strength (MPa)	Tensile Strength (MPa)	Uniform Elongation (%)	Total Elongation (%)
HT9, Tempered	440	620	4.5	13.6
LSM HT9	740	816	0.75	4.2
LSM HT9, annealed	425	635	1.50	7.7

## Data Availability

The data for this work is being demonstrated in the figures. Other data is available upon request.
